# Aminochrome Induces Irreversible Mitochondrial Dysfunction by Inducing Autophagy Dysfunction in Parkinson's Disease

**DOI:** 10.3389/fnins.2018.00106

**Published:** 2018-03-13

**Authors:** Juan Segura-Aguilar, Sandro Huenchuguala

**Affiliations:** ^1^Molecular and Clinical Pharmacology, Faculty of Medicine, Instituto de Ciencias Biomédicas (ICBM), University of Chile, Santiago, Chile; ^2^Departamento de Ciencias Biológicas y Químicas, Facultad de Ciencia, Universidad San Sebastián, Puerto Montt, Chile

**Keywords:** mitochondrial dysfunction, autophagy dysfunction, lysosomal dysfunction, aminochrome, dopamine, neuromelanin, parkinson's disease, neurodegeneration

## Mitochondrial dysfunction in parkinson's disease

In Parkinson's disease, mitochondrial complex I activity is diminished, and mitochondrial deoxyribonucleic acid (DNA) mutations accumulate (Zhang, 2013). The first evidence that mitochondrial dysfunction was involved in the pathogenesis of Parkinson's disease came from parkinsonism induced by the accidental exposure of drug users to 1-methyl-4-phenyl-1,2,3,4-tetrahydropyridine (MPTP), an inhibitor of the mitochondrial complex I of the electron transport chain (Langton et al., 1983; Esteves et al., 2011). Parker et al. (1989) found a significant reduction in the activity of complex I in platelet mitochondria purified from patients with idiopathic Parkinson's disease (Esteves et al., 2011). Further evidence for mitochondrial dysfunction in Parkinson's disease arose from studies developed in the *substantia nigra* of postmortem brains of patients with the disease, which showed deficiency of complex-I activity (Shapira et al., 1990; Esteves et al., 2011). Genes associated with familial form of the disease has been reported such as pink1, parkin, DJ-1, and CHCHD2 (Kazlauskaite and Muqit, 2015; Meng et al., 2017).

## Autophagy

Macroautophagy is the most widely studied type of autophagy, where vacuoles with double membranes form, surround cellular elements (such as proteins, lipids, and organelles), and fuse with lysosomes, the enzymes of which degrade the autophagic cargo. Autophagy is controlled by proteins that are encoded by autophagy-related genes (ATGs), ATG1–ATG35. These proteins are organized into complexes that mediate the following steps in the autophagic process: initiation, elongation, maturation, and fusion and degradation (Tan et al., 2014). The pathways that control autophagy are primarily mTOR-dependent and mTOR-independent (mTOR: the mechanistic target of rapamycin, a serine/threonine kinase). mTOR is primarily an inhibitory signal, which participates upstream of the ATG proteins. In the mTOR-independent pathway the autophagy can be directly activated by AMPK [adenosine monophosphate (AMP)-activated protein kinase], leading to direct phosphorylation of ULK1 (serine-threonine-protein kinase that is encoded by the ULK1 gene) and beclin-1 (Tan et al., 2014).

## Mitophagy

The degradation of mitochondria damaged by the autophagic pathway is known as mitophagy and constitutes one of the main mechanisms of cellular homeostasis (Zhang, 2013; Brady and Brady, 2016). Mitochondrial damage causes a decrease in mitochondrial membrane potential or an increase in mitochondrial fission, and both situations activate mitophagy (Brady and Brady, 2016). There are multiple mechanisms by which mitochondria are targeted for degradation in autophagosomes, but the best understood are the pathways of mitophagy induced by PINK/Parkin and BNIP3 (BCL2/adenovirus E1B 19 kDa protein-interacting protein 3), and NIX-dependent mitophagy (Nix: also known as BNIP3L, a BH3-only protein of the BCL-2 pro-apoptotic family). The mitochondrial protein PINK1 [phosphatase and tensin homolog (PTEN)-induced putative kinase 1], a serine-threonine kinase, is unstable due to presenilin-associated rhomboid-like protease activities (PARL). The decrease in mitochondrial membrane potential inhibits PINK1 degradation by PARL. In response to mitochondrial depolarization, PINK stabilizes, and accumulates in the outer mitochondrial membrane (OMM), where it phosphorylates ubiquitin in mitochondrial proteins to recruit autophagic cargo adapters, such as OPTN (Optineurin) and NDP52 (Nuclear dot protein 52 kDa), which directly bind to light chain 3 (LC3) in the autophagosome leading to degradation of mitochondria within autophagolysosomes (Springer and Macleod, 2016). PINK1 also recruits the E3 ubiquitin ligase Parkin and ubiquitin-specific substrates in the OMM, including VDAC (voltage-dependent anion-selective channel), Miro, and Mitofusin-2 to amplify the signal initiated by PINK (Springer and Macleod, 2016). Mutations in PARK2 (Parkin) and PARK6 (PINK1) have been independently linked to familial cases of Parkinson's disease, associating defects in mitophagy with the degeneration of dopaminergic neurons, a major feature of Parkinson's disease (Pikrell and Youle, 2015; Springer and Macleod, 2016).

The clearance of mitochondria damaged by mitophagy prevents the accumulation of dysfunctional mitochondria and can also induce mitochondrial biogenesis, increasing cell survival. On the contrary, the decrease in mitophagy occurring during aging, for example, prevents both the removal of damaged mitochondria and alters mitochondrial biogenesis, which causes the progressive accumulation of dysfunctional mitochondria (Zhang, 2013; Palikaras et al., 2015).

The dysfunction of the autophagic/lysosomal pathway is associated with mitochondrial dysfunction, which may be due to the decrease in the autophagic degradation of the dysfunctional mitochondria. For example, Wu et al. (2009) reported that deletion of an ATG7 autophagic protein in mice causes mitochondrial dysfunction, characterized by decreased mitochondrial oxygen uptake rate and increased levels of reactive oxygen species in muscle fibers and pancreatic beta cells. On the other hand, lysosomal storage disorders are associated with mitochondrial dysfunctions, which include changes in mitochondrial morphology, decreased mitochondrial membrane potential, decreased ATP production, and increased generation of reactive oxygen species (De la Mata et al., 2016; Plotegher and Dushen, 2017). The loss of lysosomal glucocerebrosidase enzyme activity causes lysosomal dysfunction (Bae et al., 2015). In turn, glucocerebrosidase deficiency is associated with mitochondrial dysfunction (Gregg and Schapira, 2016). Mitochondrial dysfunction may also induce lysosomal dysfunction, resulting in a vicious circle (Plotegher and Dushen, 2017). In the context of Parkinson's disease, mitochondrial dysfunction and lysosomal dysfunction may be connected by the oxidation of dopamine. In fact, it has recently been reported that dopamine oxidation mediates mitochondrial and lysosomal dysfunction in both sporadic and familial Parkinson's disease (Burbulla et al., 2017).

## Dopamine oxidation to neuromelanin

Dopamine oxidation to neuromelanin is a sequential pathway where several ortho(*o)*-quinones are formed: dopamine → dopamine *o*-quinone → aminochrome → 5,6-indolequinone → neuromelanin. Dopamine *o*-quinone is converted to aminochrome with a constant rate of 0.15 ^s−1^ at physiological pH (Tse et al., 1976). Oxidation of dopamine with tyrosinase to dopamine *o*-quinone in the presence of mitochondria induce the formation adduct with a long list of proteins but in the same study incubation of SH-SY5Y cells with dopamine only few adducts were detected, questioning the feasibility of dopamine *o*-quinone to be responsible for dopamine neurotoxicity (Van Laar et al., 2009). Dopaminochrome, which structure has not be determined, induces adducts with alpha-synuclein (Norris et al., 2005) and 5,6-indolequinone induced adducts with alpha synuclein (Bisaglia et al., 2007). Aminochrome is the most stable *o*-quinone.

## Aminochrome and mitochondrial dysfunction

**Figure 1:**
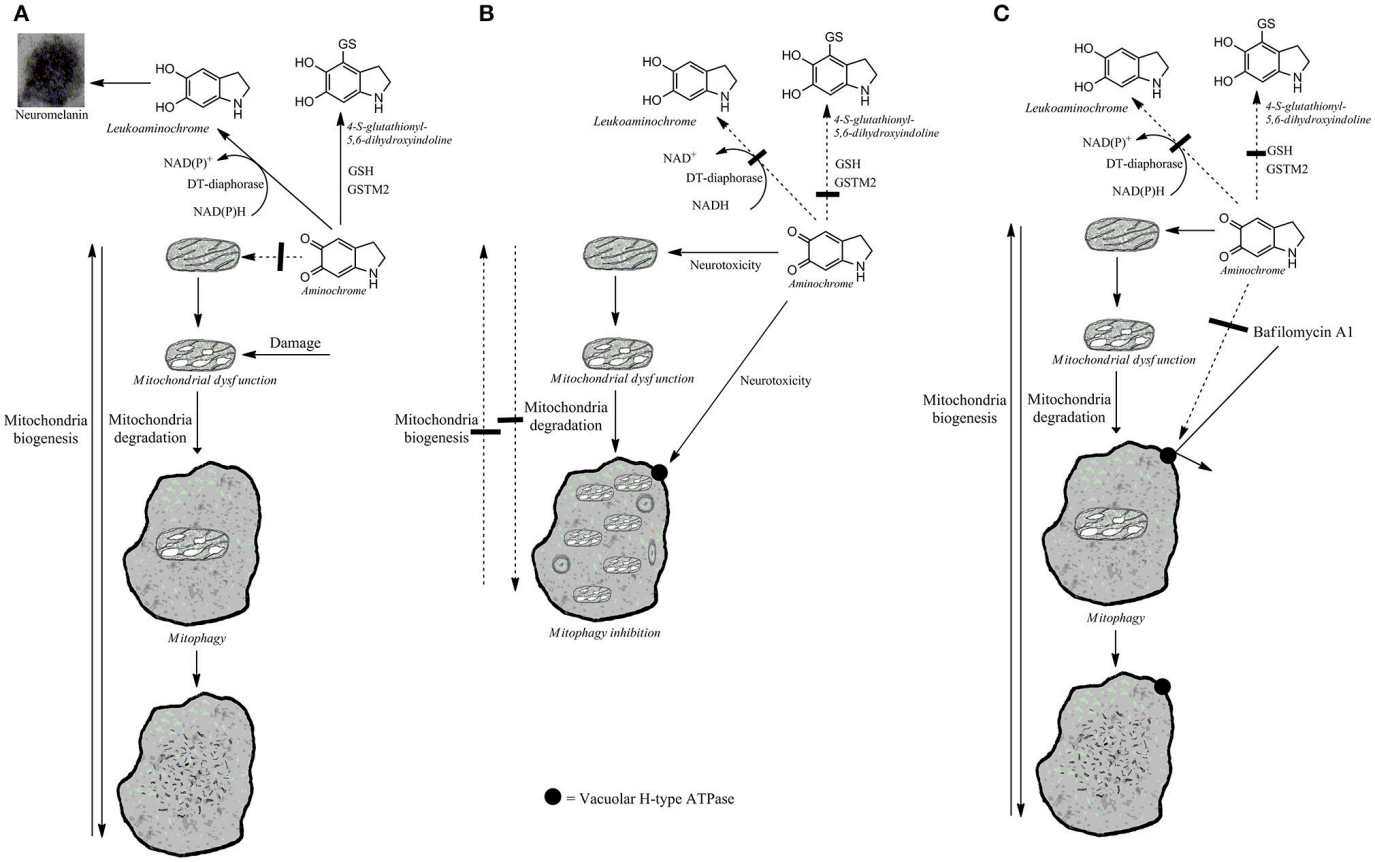
Possible aminochrome effects on mitophagy. **(A)** Damaged mitochondria are recycled by mitophagy when the protective enzymes, DT-diaphorase, and GSTM2 prevent aminochrome-induced mitochondrial and lysosomal dysfunction. The two-electron reduction of aminochrome by DT-diaphorase to leukoaminochrome prevents aminochrome neurotoxicity (Lozano et al., 2010). Aminochrome conjugation to 4-S-glutathionyl-5,6-dihydroxyindoline catalyzed by GSTM2 prevent aminochrome neurotoxicity (Huenchuguala et al., 2014). **(B)** Aminochrome induces both mitochondrial and lysosomal protein degradation dysfunction when DT-diaphorase or GSTM2 enzymes are inhibited. Damaged mitochondria cannot be recycled by mitophagy resulting in a permanent mitochondrial dysfunction. **(C)** The reversible binding of bafilomycin to vacuolar H-type ATPase localized in lysosome membrane prevents aminochrome-induced lysosomal dysfunction, allowing the recycling of aminochrome-damaged mitochondria.

For a long time, it has been accepted that mitochondrial dysfunction is involved in the degeneration of the nigrostriatal neurons containing neuromelanin in Parkinson's disease, but the question of what induces mitochondrial dysfunction inside of dopaminergic neurons containing neuromelanin remains. A possible candidate is aminochrome, an *o*-quinone formed during dopamine oxidation to neuromelanin (Segura-Aguilar et al., 2014, 2016; Herrera et al., 2017; Segura-Aguilar, 2017a,b). Aminochrome is an endogenous neurotoxin that induces mitochondrial dysfunction by inhibiting complex I and decreasing ATP levels in different cell lines (Arriagada et al., 2004; Aguirre et al., 2012; Huenchuguala et al., 2017). Aminochrome induces mitochondrial dysfunction in rat brain, resulting in a significant decrease in ATP levels, which explains a significant the decrease in dopamine release and amount of synaptic vesicles at the synaptic cleft. Both the axonal transport of neurotransmitter vesicles to the terminals and dopamine release require ATP (Herrera et al., 2016). Interestingly, aminochrome also induces other mechanisms related to the degeneration of dopaminergic neurons containing neuromelanin, such as protein degradation dysfunction of both lysosomal and proteasomal systems (Zafar et al., 2006; Huenchuguala et al., 2014); aggregation of alpha-synuclein to neurotoxic oligomers (Muñoz et al., 2015); neuroinflammation (Santos et al., 2017); oxidative (Arriagada et al., 2004); and endoplasmic reticulum stress (Xiong et al., 2014). The oxidation of dopamine to neuromelanin is a normal pathway, because healthy seniors have intact dopaminergic neurons containing neuromelanin (Zecca et al., 2002; Zucca et al., 2017). A relationship between neuromelanin content and loss of dopaminergic neurons containing neuromelanin has been reported. The level of neuromelanin in substantia nigra pars compacta was 10 times higher than in ventral tegmental area of control subjects. The loss of dopaminergic neurons containing neuromelanin in Parkinson's disease patients was found to be 47% in comparison 0% in ventral tegmental area (Schwarz et al., 2017). The 10-fold lower amount of neuromelanin in ventral tegmental area results in lower amount of aminochrome, explaining why these neurons were intact in this study (Schwarz et al., 2017). Neuromelanin formation depends on VMAT-2 expression because dopamine is complete stable inside of monoaminergic vesicles where the pH is around 5.3 due to dopamine uptake is coupled to an ATPase proton pump (Sulzer et al., 2000; Herrera et al., 2017; Segura-Aguilar, 2017a,b). An inverse relationship between VMAT-2 expression level and neuromelanin content in human midbrain dopamine neurons has been reported (Liang et al., 2014). The reason why aminochrome is not neurotoxic in dopaminergic neurons containing neuromelanin in healthy seniors is because the enzymes DT-diaphorase and glutathione transferase M2-2 (GSTM2) prevent aminochrome neurotoxicity (Lozano et al., 2010; Huenchuguala et al., 2014, 2016; Segura-Aguilar, 2015, 2017a; Herrera-Soto et al., 2017; Muñoz and Segura-Aguilar, 2017) (Figure 1). DT-diaphorase is expressed both in dopaminergic neurons and astrocytes, but GSTM2 is only expressed in astrocytes. However, GSTM2 protects both astrocytes and dopaminergic neurons against aminochrome neurotoxicity because astrocytes secrete GSTM2 and dopaminergic neurons internalize GSTM2 into the cytosol (Cuevas et al., 2015; Segura-Aguilar et al., 2016; Segura-Aguilar, 2017b,c). The question is why GSTM2 and DT-diaphorase are not protecting dopaminergic neurons containing neuromelanin in Parkinson's disease patients. A possible explanation is that an over production of aminochrome surpass the enzyme capacity (Km) to prevent its neurotoxic effects or for some unknown reason these enzyme are down regulated or inhibited.

## The importance of mitophagy in preventing mitochondrial dysfunction

In general, mitochondrial dysfunction activates mitophagy as a defense mechanism to remove damaged mitochondria (Brady and Brady, 2016). Mitophagy seems to play a key homeostatic role in mitochondrial quality control (Tan and Wong, 2017). It has been proposed that age-dependent deterioration of mitophagy both inhibits the removal of damaged mitochondria and impairs mitochondrial biogenesis (Palikaras et al., 2017). The problem is when mitochondrial dysfunction is induced by a neurotoxin that induces both mitochondrial and autophagy dysfunction. Aminochrome-induced mitochondrial dysfunction is irreversible because aminochrome also induces autophagy dysfunction by preventing the fusion between autophagy vacuoles and lysosome dysfunction by increasing their pH (Huenchuguala et al., 2014). Recently, we have demonstrated that mitophagy plays a key role in reversing aminochrome-induced mitochondrial dysfunction (Huenchuguala et al., 2017). The pre-incubation of cells with bafilomycin A1, a reversible inhibitor of lysosomal vacuolar-type H^+^-ATPase, before the incubation with aminochrome restores ATP levels, mitochondrial membrane potential, and mitophagy, and decreases cell death (Huenchuguala et al., 2017). Aminochrome cannot form adduct with vacuolar-type H^+^-ATPase because bafilomycin A1 pre-incubated with the cells prevents it. Then after a while, bafilomycin dissociates from ATPase but aminochrome is not as stable to form adducts with ATPase after bafilomycin has dissociated. When aminochrome is prevented from binding to lysosomal vacuolar-type H^+^-ATPase it can bind other proteins or can be reduced by flavoenzymes. These experiments support (i) the enormous importance of mitophagy in preventing mitochondrial dysfunction and (ii) the irreversible neurotoxic action of aminochrome, as a consequence of its ability to be neurotoxic by inducing both mitochondria and mitophagy dysfunction (Figure 1).

## Conclusion

Mitochondrial dysfunction seems to play an important role in the loss of dopaminergic neurons containing neuromelanin in the nigrostriatal system in Parkinson's disease. The accurate removal of dysfunctional mitochondria by mitophagy is essential for keeping control over normal mitochondrial function in neurons. The endogenous neurotoxin aminochrome induces an irreversible mitochondrial dysfunction because it induces both mitochondrial and lysosomal protein degradation dysfunction. However, when bafilomycin A1 prevented aminochrome-dependent lysosomal dysfunction, the normal mitochondrial function was recovered, highlighting the essential role of the lysosomal protein degradation system in the prevention of mitochondrial dysfunction in Parkinson's disease.

## Author contributions

JS-A: design and write the paper; SH: write a part of the paper.

### Conflict of interest statement

The authors declare that the research was conducted in the absence of any commercial or financial relationships that could be construed as a potential conflict of interest.
